# The Pathobiology of Interleukin 11 in Mammalian Disease is Likely Explained by its Essential Evolutionary Role for Fin Regeneration

**DOI:** 10.1007/s12265-022-10351-9

**Published:** 2023-01-11

**Authors:** Stuart A. Cook

**Affiliations:** 1 Cardiovascular and Metabolic Disorders Program, Duke-National University of Singapore Medical School, Singapore, Singapore; 2 National Heart Research Institute Singapore, National Heart Centre Singapore, Singapore, Singapore; 3 Institute of Medical Sciences, Hammersmith Hospital Campus, London, UK

**Keywords:** regeneration, IL-11, fibrosis, EMT

## Abstract

Recent studies have shown IL11 to be pro-fibrotic, pro-inflammatory and anti-regenerative in heart, liver, lung and kidney disease in mice and humans. However, data also show that IL11 is specifically required for appendage regeneration following trauma in some species. In fish, tadpoles and axolotl, IL11 is uniquely upregulated in the regenerative organ, the blastema, following loss of fin, tail or limb. In this short essay I suggest that the pathobiology of IL11 in mammals is rooted in its deep evolutionary role for epimorphic appendage regeneration. In both blastema formation and mammalian disease there is robust IL11-driven fibroblast activation, extracellular matrix production, inflammation and epithelial cell dedifferentiation. While these cellular processes are critical for regeneration in lower species they cause organ failure in mammals. This hypothesis, if correct, may explain the apparent redundancy of IL11 for human health and suggest IL11 as a therapeutic target.

**Graphical Abstract:**

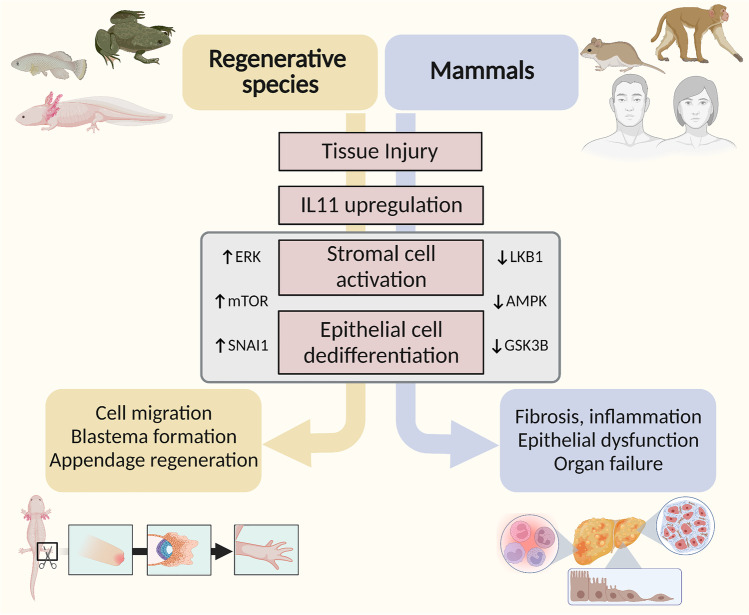

Interleukin 11 (IL11) is a little-studied member of the IL6 family of cytokines that has recently been shown to have pro-fibrotic, pro-inflammatory and anti-regenerative properties [1]. IL11 is upregulated in a range of fibro-inflammatory diseases, first identified in cardiorenal disease and contributes to disease pathology. IL11 expression in disease is apparent in damaged epithelial cells and activated fibroblasts that secrete IL11 as an alarmin-like response when exposed to infective, environmental, metabolic or genetic stressors.

IL11 secreted from damaged cells acts in autocrine and paracrine to transiently activate STAT3 leading to pro-inflammatory gene expression. In a more sustained fashion, IL11 increases the activity of ERK and p90RSK, which act together to dually phosphorylate and inactivate both LKB1 (STK11) and GSK3β leading to an mTOR- and SNAI1-driven program of epithelial-to-mesenchymal transition (EMT) [2]. This causes epithelial cell dedifferentiation and loss of specialised polarised functions as well as fibroblast-to-myofibroblast transformation. In disease, this leads to (1) loss of homeostatic epithelial cell functions, (2) fibrosis and (3) stromal-driven inflammation.

IL11RA receptors are most highly expressed on stromal and epithelial cells, whereas IL6R is more specific for immune cells. Unlike IL6, IL11 is barely detectable in health. Loss of IL11 signalling in humans, mice, tadpoles and fish has limited effects in health whereas inhibition of IL6, STAT3 or gp130 is very detrimental [1, 3, 4]. In the general population, *IL11* and *IL11RA* have accumulated loss-of-function (LoF) mutations in the absence of selective constraint, which suggests these genes are unimportant for reproductive health. This said, human knockouts for *IL11RA* can have mild skull and tooth abnormalities, and female mouse *Il11ra1* knockouts are infertile. LoF mutations in other IL6 family members or their receptors are strongly selected against (https://gnomad.broadinstitute.org/). This begs the following questions: why is IL11 upregulated in damaged mammalian tissues to cause disease, why does IL11 appear redundant in healthy humans, why does IL11 initiate a program of EMT and are these matters related?

It has been said that ‘nothing in biology makes sense except in the light of evolution’ (Theodosius Dobzhansky, 1973). IL11 is thought to have evolved in fish some 450 million years ago, and here I explore IL11 biology in an evolutionary context, in fish and tadpoles, with the idea that this might shed light on its detrimental effects in human disease.

In the zebrafish, *il11* is strongly linked with organ regeneration, and *il11* is specifically upregulated at sites of trauma across fish species (e.g. lungfish, killifish). Genetic loss of function of either *Il11ra* or *il11a* in the zebrafish prevents regeneration of the larval fin fold, adult heart, caudal fin and scales [4]. This effect is accompanied by impaired activation of critical pro-regeneration genes (e.g. *devoid of blastema*) along with reduced polarised cell dedifferentiation. Regeneration in the zebrafish is characterised by fibroblast activation and extracellular matrix production, without which heart regeneration is impaired [5].

*Xenopus laevis* tadpoles regenerate their tails following amputation, and this effect is associated with the unique upregulation of *il11* in the regeneration bud at the site of injury. Knockdown of *il11ra.L* or *il11* in *Xenopus* tadpoles prevents tail regeneration, which is associated with reduced neuron, mesenchymal and muscle cell dedifferentiation and reduced cell migration. As for zebrafish, inhibition of il11 signalling in *Xenopus* does not affect normal development, and survival after trauma is normal. In the axolotl, IL11 is one of the most upregulated genes at the site of trauma following limb amputation.

Epimorphic appendage regeneration following traumatic fin, tail or limb amputation in fish, tadpoles and axolotl has a deep evolutionary origin and an absolute requirement for the regenerative organ—the blastema, where IL11 is distinctly expressed [6]. The blastema is a heterogeneous mass of migrating immune cells, activated fibroblasts and dedifferentiated bone, muscle, neuron and endothelial cells that forms at the site of injury. Cells in the mesenchyme-rich blastema proliferate and redifferentiate to generate a new appendage. Robust inflammation, immune evasion and matrix production are essential for normal blastema function. In the absence of IL11, blastema function fails and so does regeneration.

How can we reconcile these seemingly conflicting data: that IL11-driven blastema development leads to limb regeneration in lower species whereas IL11-activated processes cause fibroinflammatory diseases in mammals? On closer inspection, it is apparent that IL11 causes fibroblast activation, matrix production, inflammation and epithelial dedifferentiation (via EMT) in all species, across the evolutionary scale. In lower species, redifferentiation of the blastema resolves fibroinflammation and regenerates damaged tissues. Mammals do not generate blastemas but still activate IL11 that leads to a disorganised milieu of activated fibroblasts, inflammatory cells and dysfunctional epithelial cells (Fig. 1).

**Fig. 1:**
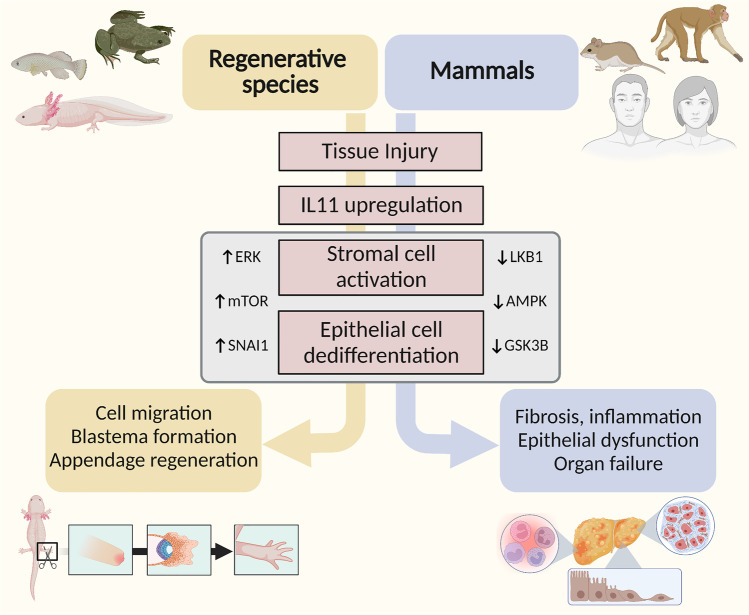
Cartoon hypothesis showing how tissue trauma across species causes IL11 upregulation that acts on both epithelial and stromal cells to activate ERK, p90RSK and mTOR while inactivating LKB1, AMPK and GSK3β. This leads to fibroblast-to-myofibroblast transformation, extracellular matrix production and inflammation as well as dedifferentiation of epithelial cells (e.g. neuronal/muscle/bone/endothelial cells). In regenerative species, IL11-stimulated cells migrate to form a blastema where they proliferate and redifferentiate to form a replacement appendage. In non-regenerative species, the activated myofibroblasts and dedifferentiated epithelial cells fail to form a blastema and are stalled in a dysfunctional state that causes tissue fibrosis, inflammation and failed epithelial repair leading to organ failure. This is evident in mouse models of heart, lung, liver and kidney failure. Created with BioRender.com on 14/12/2022

To answer the questions posed above: (1) why is IL11 upregulated in damaged mammalian tissues to cause disease? This likely reflects an unhelpful legacy of IL11’s evolutionary role—an attempt to form a blastema that fails in mammals with deleterious consequences. (2) Why does IL11 appear largely redundant in healthy humans? As humans do not undergo epimorphic regeneration, there are limited benefits of IL11 in humans. (3) Why does IL11 initiate a program of EMT across cell types? This comes back to the ancestral role of IL11 in blastema formation where EMT-enabled cell migration features prominently.

I propose that while fibrosis and inflammation are often viewed as sequelae of failed regeneration, they should instead be seen as critical, IL11-dependent components of regeneration, in lower species. In mammals, IL11 remains ‘hard-wired’ to respond to injury and continues to activate components of a deep evolutionary cellular program of repair. However, in the absence of a blastema, IL11 causes disease instead of regeneration in warm-blooded mammals. I end by suggesting IL11 as a therapeutic target as it appears to have limited homeostatic functions in humans but causes pathobiology across cellular compartments in both rare and common human diseases.

## References

[1] Cook SA, Schafer S (2020). Hiding in plain sight: interleukin-11 emerges as a master regulator of fibrosis, tissue integrity, and stromal inflammation. Annu Rev Med.

[2] Widjaja AA, Viswanathan S, Wei Ting JG, Tan J, Shekeran SG, Carling D, … Cook SA. IL11 stimulates ERK/P90RSK to inhibit LKB1/AMPK and activate mTOR initiating a mesenchymal program in stromal, epithelial, and cancer cells. iScience (2022);25(8):104806. 10.1016/j.isci.2022.104806 PMC9386112 35992082

[3] Suzuki S, Sasaki K, Fukazawa T, Kubo T (2022). Xenopus laevis il11ra.L is an experimentally proven interleukin-11 receptor component that is required for tadpole tail regeneration. Sci Reports.

[4] Allanki S, Strilic B, Scheinberger L, Onderwater YL, Marks A, Günther S, … Reischauer S. Interleukin-11 signaling promotes cellular reprogramming and limits fibrotic scarring during tissue regeneration. Sci Adv (2021);*7*(37):eabg6497. 10.1126/sciadv.abg6497 PMC8442930 34516874

[5] Hu B, Lelek S, Spanjaard B, El-Sammak H, Simões MG, Mintcheva J, … Junker JP. Origin and function of activated fibroblast states during zebrafish heart regeneration. Nat Genet 54(8):1227–1237 10.1038/s41588-022-01129-5 PMC7613248 35864193

[6] Seifert AW, Muneoka K (2018). The blastema and epimorphic regeneration in mammals. Dev Biol.

